# Complete Genome Sequence of an Aeromonas rivuli Strain Isolated from Ready-to-Eat Food

**DOI:** 10.1128/mra.01130-21

**Published:** 2022-04-20

**Authors:** Keike Schwartz, Maria Borowiak, Eckhard Strauch, Carlus Deneke, Martin Richter

**Affiliations:** a German Federal Institute for Risk Assessment, Department of Biological Safety, Berlin, Germany; University of Maryland School of Medicine

## Abstract

Aeromonads can be associated with diseases in animals and humans. Knowledge regarding Aeromonas rivuli, a species recently discovered in creek water in Germany, is still fragmentary. Here, we announce the complete genome sequence of Aeromonas rivuli strain 20-VB00005, which was recovered from ready-to-eat food.

## ANNOUNCEMENT

Bacteria of the Gram-negative genus *Aeromonas* are ubiquitously distributed in aquatic environments and are occasionally isolated from livestock (1–3). Four *Aeromonas* species are considered important opportunistic pathogens in humans, i.e., Aeromonas caviae, Aeromonas dhakensis, Aeromonas veronii, and Aeromonas hydrophila (2).

In 2019, an *Aeromonas* species isolate was recovered from pasteurized milk in German retail outlets. Investigation of the producer revealed that the milk had been contaminated by cooling water due to a defective seal in the production facility. All batches of the potentially contaminated milk were withdrawn from the market, because biochemical characterization of the isolate could not rule out a potential pathogenic *Aeromonas* isolate. For clear identification following matrix-assisted laser desorption ionization–time of flight mass spectrometry (MALDI-TOF MS)-based (Bruker Microflex LT/SH MBT) and biochemical (bioMérieux API20NE) *Aeromonas* prediagnostic analyses, the new isolate (20-VB00005) was subjected to whole-genome sequencing.

The isolate was obtained from a milk sample by cultivation on Merck GSP agar for 72 h at 25°C (4). After cultivation in tryptic soy broth for 24 h at 23°C, genomic DNA was extracted using the PureLink genomic DNA minikit (Thermo Fisher Scientific) and sequenced using Illumina and Oxford Nanopore Technologies (ONT) instruments.

An Illumina sequencing library was prepared using the Illumina DNA Prep (M) Tagmentation kit. Paired-end sequencing was carried out in 2 × 201-bp cycles on an Illumina MiSeq device using the MiSeq reagent kit v3 (600 cycles). Illumina short reads were trimmed using fastp v0.19.5 (5). After bioinformatic preprocessing, 1,889,064 high-quality reads (95.95% of bases having scores of ≥Q30) were available. An ONT sequencing library was prepared using the ONT rapid barcoding kit (SQK-RBK004) and sequenced on an ONT MinION Mk1C sequencer (running with MinKNOW v21.02.2) using an R9.4.1 (FLO-MIN106D) flow cell. ONT long reads were trimmed using Porechop v0.2.4 (https://github.com/rrwick/Porechop) and filtered using NanoFilt v2.8.0 (6). A quality check using NanoStat v1.5.0 (6) revealed 12,553 reads, with a read *N*_50_ value of 14,345 bp and a mean read quality score of 10.7. A hybrid assembly was performed using Unicycler v0.4.8 (with Pilon v1.24 polishing) (7–9), which automatically removes overlaps and circularizes and rotates/flips the assembly with a starting point at the *dnaA* gene.

Similarity calculation between the resulting 20-VB00005 genome (Table 1) and the DSM 22539^T^ genome using the OrthoANIu algorithm (10) revealed an average nucleotide identity (ANI) value of 98.12 (>95)%, assigning the new isolate to the recently discovered, presumed environmental species Aeromonas rivuli (11, 12).

**TABLE 1:** Genomic features and PGAP annotation data for the Aeromonas rivuli food strain 20-VB00005

Feature	Finding for Aeromonas rivuli 20-VB00005
Genome organization	1 chromosome (circular)
Genome size (bp)	4,357,928
GC content (%)	59.9
No. of CDSs	
Total	3,863
Protein CDSs	3,829
Pseudogenes	34
No. of RNA genes	
Total	157
rRNAs	31
tRNAs	122
Noncoding RNAs	4

**FIG 1:**
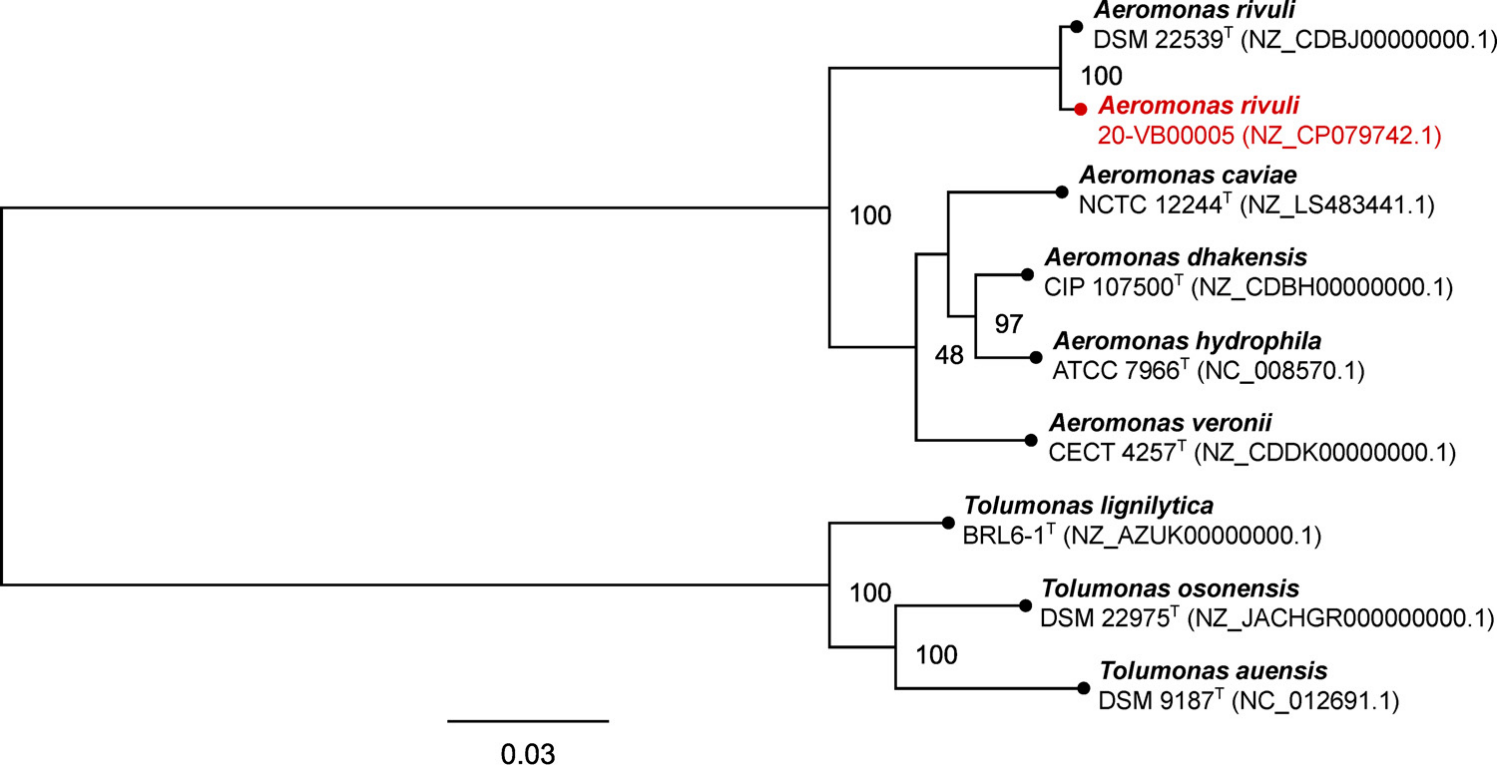
Best-scoring maximum likelihood tree based on a comparison of the amino acid sequences of 107 essential single-copy core genes of the Aeromonas rivuli food strain 20-VB00005, the environmental Aeromonas rivuli type strain, major human-pathogenic *Aeromonas* spp., and closely related *Tolumonas* spp. using bcgTree v1.1.0. The tree was visualized using Geneious Prime v2020.2.2 (Biomatters), rooted using the *Tolumonas* species node as an outgroup, and finalized using Inkscape v0.92.4. Numbers to the right of nodes designate bootstrap support values (*n* = 100 bootstrap replicates). Superscript T indicates type strain. Numbers in parentheses are NCBI RefSeq accession numbers; protein fasta sequences deposited in the NCBI RefSeq database served as bcgTree input files. The scale bar represents the number of amino acid substitutions per site.

The A. rivuli 20-VB00005 genome was annotated using PGAP v5.3 (https://www.ncbi.nlm.nih.gov/genome/annotation_prok) (Table 1) and compared to PGAP-annotated *Aeromonas* sp. and *Tolumonas* sp. type strain genomes from the NCBI Reference Sequence (RefSeq) database. Using the translated coding sequences (CDSs) provided by NCBI, the phylogeny of the different *Aeromonadaceae* strains was inferred through comparison of the amino acid sequences of 107 single-copy core genes with bcgTree v1.1.0 (13). For all software mentioned in this report, default parameters were used unless specified otherwise. The maximum likelihood tree (Fig. 1) reveals that the food strain 20-VB00005 is closely related to the environmental A. rivuli strain DSM 22539^T^, while strains of known major human-pathogenic *Aeromonas* species form a separate subcluster. The pathogenic potential of the *Aeromonas* strain discovered in pasteurized milk is currently not assessable.

### Data availability.

Sequencing reads for 20-VB00005 were deposited in the NCBI SRA (accession numbers SRX11480609 [Illumina data] and SRX11480610 [ONT data]). The complete genome sequence is available at NCBI RefSeq (accession number NZ_CP079742).
